# Infectious disease management in natural disasters

**DOI:** 10.1017/ash.2026.10843

**Published:** 2026-09-10

**Authors:** Kelsey G. Trulik, David J. Weber, Stephen Y. Liang, David K. Warren, Trini A. Mathew

**Affiliations:** 1 Department of Emergency Medicine, Emory University School of Medicine, Atlanta, GA, USA; 2 Division of Infectious Diseases, The University of North Carolina at Chapel Hill School of Medicine, Chapel Hill, NC, USA; 3 Division of Infectious Diseases, Department of Medicine and Department of Emergency Medicine, Washington University School of Medicine, St. Louis, MO, USA; 4 Division of Infectious Diseases, Department of Internal Medicine, University of Nebraska Medical Center, Omaha, NE, USA; 5 Department of Internal Medicine, Wayne State University School of Medicine, Detroit, MI, USA

## Abstract

During humanitarian emergencies, infectious diseases may present during the acute, subacute, and/or recovery phase, requiring focused surveillance and management. Effective response depends on early identification, appropriate isolation, and timely public health notification. Prevention strategies addressing shelter, potable water, sanitation, vector control, nutrition, immunization, and health education are essential in eliminating or reducing the likelihood of outbreaks. Disaster-related infectious disease management requires an integrated, systems-based approach encompassing identification, surveillance, clinical care, public health, prevention, and infrastructure resilience. This manuscript provides a narrative review of relevant sources to guide facilities in identifying and managing key infectious diseases during disasters with a focus on United States (US) based recommendations. Resources include best practices from the American College of Physicians (ACEP), US Centers for Disease Control and Prevention (CDC), US Federal Emergency Management Agency (FEMA), World Health Organization (WHO), and US National Emerging Special Pathogens Training and Education Center (NETEC).

## Introduction

Natural disasters are increasing globally. The United Nations Office for Disaster Risk Reduction defines disasters as “a serious disruption of the functioning of a community or a society at any scale due to hazardous events interacting with conditions of exposure, vulnerability and capacity, leading to one or more of the following: human, material, economic and environmental losses and impacts.”^1^ The open-access Emergency Events Database (EM-DAT) maintains a living record of global disasters and their impact dating back more than a century. From 1900 to 2000, the prevalence of disaster rose exponentially and has since plateaued.^2^ To meet inclusion in this database, a disaster must have met one or more of the following criteria: (1) at least ten deaths (dead or missing), (2) at least 100 affected people, or (3) a call for international assistance or an emergency declaration.^3^ Extreme weather events and floods are the most reported natural disasters worldwide and reflect the greatest absolute and relative change since 1900.^2^ According to the United Nations Framework Convention on Climate Change, climate change and weather-related disasters increased five-fold and economic losses increased seven-fold between 1970 and 2019.^4,5^ Fortunately, with improvements in disaster management and early warning systems, the number of disaster-related deaths has decreased three-fold.^5^

### Impact of natural disasters

**Figure 1.**
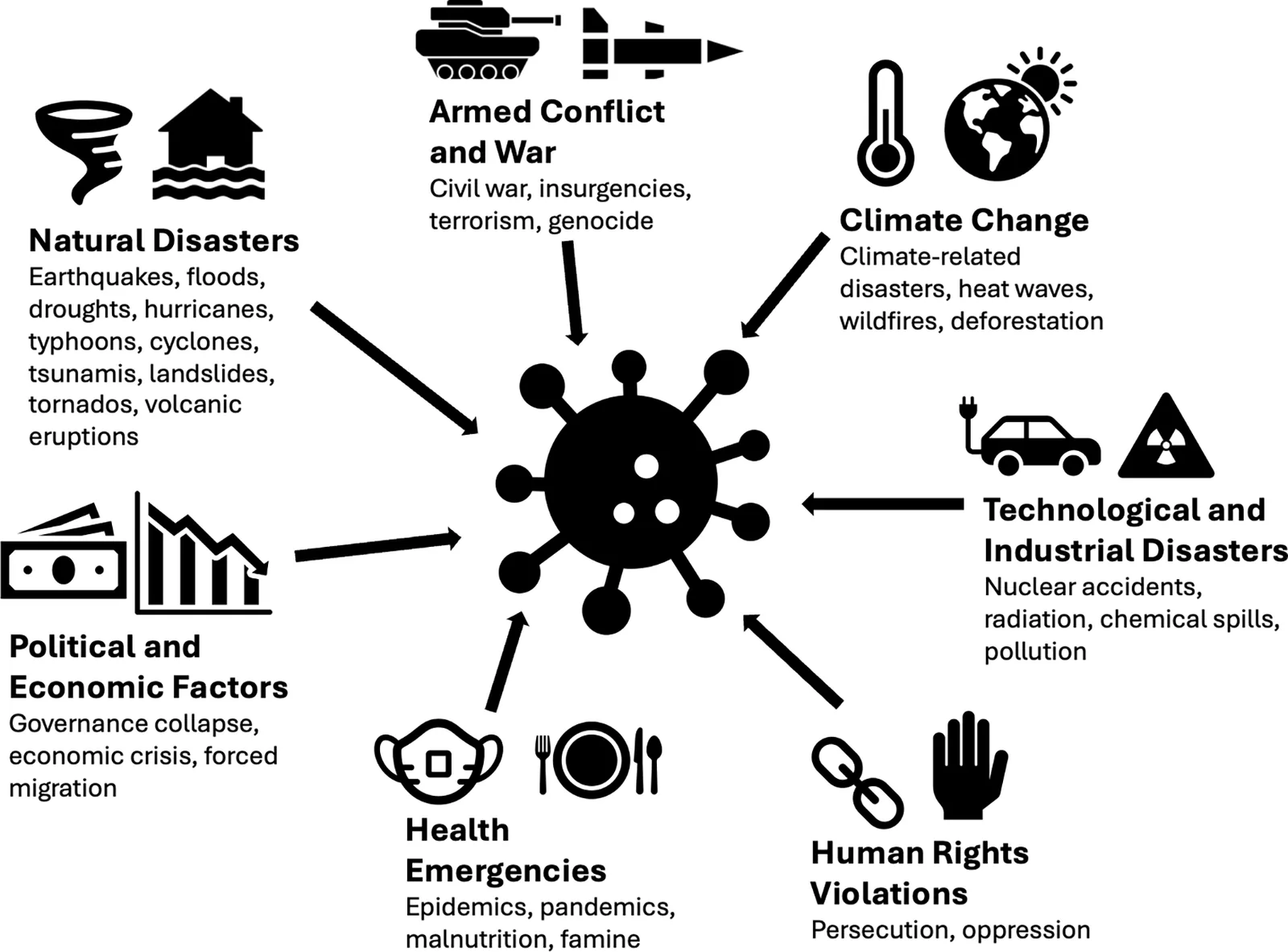
Contributors to humanitarian emergencies leading to infectious and non-infectious diseases.

Three hundred and ninety-three natural hazard-related disasters were reported in 2024, an increase from the preceding two decades. The year 2024 was marked by extreme temperature events in Asia resulting in thousands of deaths, severe droughts in Africa affecting over 25 million people, and devastating tropical storms in the US incurring cumulative damages more than US ＄100 billion.^6^ In total, these disasters led to 16,753 deaths and impacted 167.2 million people. An estimated 96.1 million people are living in acute food insecurity driven by these weather extremes. Worldwide, economic losses totaled US ＄241.95 billion. Although high-income countries account for most absolute economic losses, the human toll of these disasters has been disproportionately borne by low- and middle-income countries.^3^ Many factors during humanitarian emergencies influence and contribute to non-infectious and infectious diseases (Figure 1).

### Multi-agency coordination during disasters in the US

Disaster recovery is a complex, highly interdependent process shaped by the nuances of humanitarian emergencies. In the US, the Federal Emergency Management Agency (FEMA) provides a National Disaster Recovery Framework that outlines strategies for rebuilding and strengthening disaster-affected communities.^7^ Predisaster planning and preparedness are essential to mitigate the impact of disasters on communities. State and local health departments, emergency management agencies, and healthcare facilities play key roles in the response to natural disasters. Since July 2024, regulatory and accreditation agencies require US hospitals to have programs in place for managing high consequence infectious diseases (eg, Ebola) and other special pathogens (eg, Mpox).^8^ When disasters strike, the immediate response focuses on lifesaving and life-sustaining actions, as well as safeguarding critical community systems: water, sanitation, shelter, food, energy, communication, transportation, and safety. Therefore, building resilience across the disaster continuum is essential to enabling communities to better prepare for, withstand, and recover from future disasters.^7^

### Best practices to prevent infectious diseases

In this review, we focus on the response to and management of infectious diseases during and after natural disasters. The primary intended audiences are frontline clinicians (eg, emergency physicians and other healthcare personnel in emergency departments) as well as infectious diseases physicians, healthcare/hospital epidemiologists, infection preventionists, and prehospital emergency response teams. Although this overview is focused on technical aspects of infectious diseases management after a natural disaster, the resilience of the survivors impacted and that of the first responders, healthcare and public health professionals, and government and community leaders also factor substantially in a successful response.

## Injuries and conditions contributing to infections

Individuals may sustain physical injuries and environmental exposures during a disaster requiring urgent evaluation and management (Table 1). Suboptimal or lack of medical care can predispose to infection. For example, lacerations/abrasions exposed to storm surge water can lead to serious infections with *Vibrio* spp., thermal burns can become superinfected by bacteria and fungi, and near drowning with aspiration of contaminated water can lead to pneumonia.

**Table 1.** Outline of non-infectious injuries and diseases during and post-natural disasters that may lead to superimposed or subsequent infections Table 1 long description.

Category	Definition	Information	Disaster-related examples
Drowning	“Drowning is the process of experiencing respiratory impairment from submersion/immersion in liquid”^9^	– Estimated 300,000 drowning related deaths annually – Drowning rates are highest amongst children less than 5 years old – Drowning related deaths are three times greater in low- and middle-income countries – Drowning accounts for 75% of deaths in flood disasters^9,10^	Riptides Car immersion Home water intrusion Storm surge
Burn injuries	“A burn is an injury to the skin or other organic tissue primarily caused by heat or due to radiation, radioactivity, electricity, friction or contact with chemicals”^11^	– Estimated 180,000 burn related deaths annually – Non-fatal burn injuries are a leading cause of morbidity – Burn related child deaths are seven times greater in LMICs^11^	Inhalation Rupture of gas lines Downed electrical lines Cooking on gas grills
Blunt force trauma	“Direct contact of a blunt object with a body”^12^	– Sixth leading cause of death worldwide – Traumatic rhabdomyolysis, crush syndrome, is a major cause of mortality in disaster victims^12,13^	Trees falling Flying objects due to wind Crush injuries
Bites	“Injury caused by the mouth and teeth of an animal (including humans). There may be bruising, deep anatomic structure disruption, and introduction of infectious agents”^14^	– Estimated 5 million people are bitten by snakes annually, with ~50% of them ending up with envenomation – Bites by venomous snakes can result in paralysis, bleeding disorders, kidney failure, and tissue damage^15^ – 94,000–125,000 deaths are due to envenomation-related complications annually^16^	Snakes (water moccasins, coral snakes, copper heads) Alligators/Crocodiles Dogs/Cats
Carbon monoxide poisoning	“Inhalation of carbon monoxide, an odorless, colorless gas”^17^	– The most common symptoms are headache, dizziness, weakness, upset stomach, vomiting, chest pain, and confusion – Severe symptoms include malaise, shortness of breath, nausea, ataxia, altered mental status, loss of consciousness, coma, and death^17,18^	Indoor heaters Portable generators Cars when run in closed spaces (eg, garage) Cooking on gas grills in enclosed spaces
Electrocution	“Death caused by an electrical injury”^19^	– Injuries from electrical shock may not be visible – Higher voltage injuries cause additional injuries including severe muscular contractions, nerve damage, cardiac arrest, severe burns, and death^19^	Downed wires Exposed power lines
Medical	“Conditions that last 1 year or more and require ongoing medical attention or limit activities of daily living or both”^20^	– Roughly 75% of American adults have at least one chronic medical condition – Disasters disrupt management of chronic diseases by restricting access to healthcare facilities, specialists, and medications^20,21^	Heart attacks Asthma Cancer Diabetes Kidney disease Need for power for certain medical devices
Psychiatric	“Clinically significant disturbance in an individual’s cognition, emotional regulation or behavior”^22^	– Estimated 70% of people globally will experience a traumatic event during their lifetime – Estimated 4% of the world population has experienced PTSD^22,23^	PTSD Depression Suicide Violence

Disruption of critical public health infrastructure and community systems creates conditions conducive for infectious diseases. Survivors may develop bacterial infections from poor wound care due to soil, water, and environmental pathogens. Community outbreaks may occur in emergency shelters where displaced persons reside near another and have limited access to water, sanitation, and personal hygiene. These infections may include respiratory diseases transmitted through aerosols or droplets or gastrointestinal diseases transmitted person-to-person through fecal-oral spread or ingestion of contaminated food or water. Proximity can also lead to infestations, such as lice and scabies. Within hospitals, the risk of healthcare associated infection may increase due to inadequate water management and breakdown of facility infrastructure, including legionellosis and fungal infections.^24,25^

## Infections during phases of disaster response

**Figure 2.**
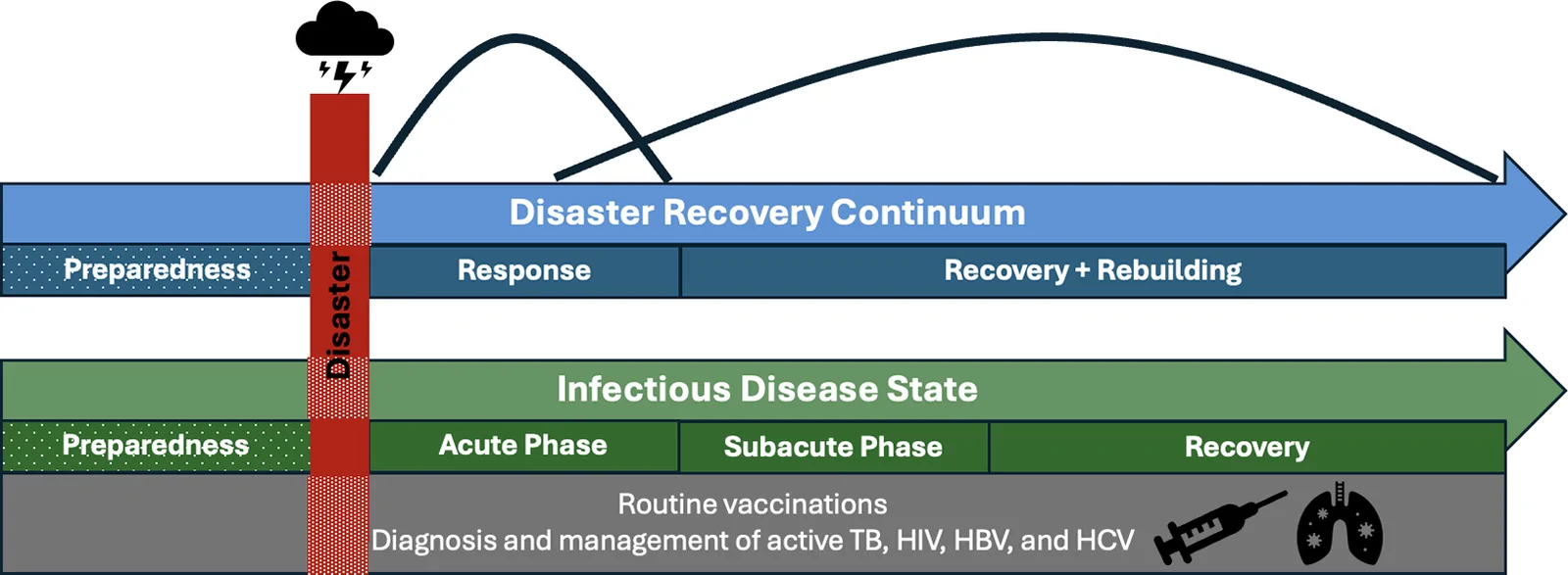
Phases of disasters and response and management of infectious diseases.

Disaster planning and preparedness to manage infectious diseases should ideally take place well in advance of a natural disaster and directly impact the acute, subacute, and recovery phases after a disaster. The approach to managing infectious diseases can vary in the acute, subacute, and recovery phases following a natural disaster (Figure 2).^26,27^ During the acute phase, infections with short incubation periods (a few days to a few weeks) are likely to be encountered, including foodborne disease, other gastrointestinal infections (eg, norovirus after Hurricane Katrina; hepatitis A outbreaks following floods), respiratory infections, and skin and soft tissue infections.^28–32^ In the subacute phase, infections with longer incubation periods (weeks to months) are more common, often related to contact with other survivors and even responders. Following the 2010 Haiti earthquake, a widespread community outbreak of cholera was linked to international rescue workers.^33^ Scabies infestation was encountered among earthquake survivors at a camp in Nepal.^34^ During the recovery phase, infections with lengthier incubation periods are more likely. Chronic or indolent infections may be exacerbated by disruptions in ongoing medical treatment, including tuberculosis (TB) (both preexisting and new infection), non-tuberculous mycobacteria, hepatitis B virus (HBV), hepatitis C virus (HCV), and human immunodeficiency virus (HIV).^35–38^ In the case of HIV, lapses in adherence to antiviral therapy may contribute to the spread of HIV among sexually active adults.^39,40^ Infectious diseases after natural disasters vary by setting, route of transmission, and isolation precautions needed to interrupt transmission (Table 2).^41^ The appropriate transmission-based precautions should be initiated at the earliest opportunity to prevent further spread of disease. Standard precautions are used for all patient care and refer to appropriate hand hygiene, use of personal protective equipment (PPE), respiratory hygiene, cough etiquette, cleaning and/or disinfection of equipment and environment, and safe injection practices.^42^ Contact precautions include the use of gloves and gowns. Droplet precautions include the use of nose and mouth covering (and may require face shields/goggles). Airborne precautions include the use of a fit-tested N95 or other respirator and closed doors.^43^

**Table 2.**
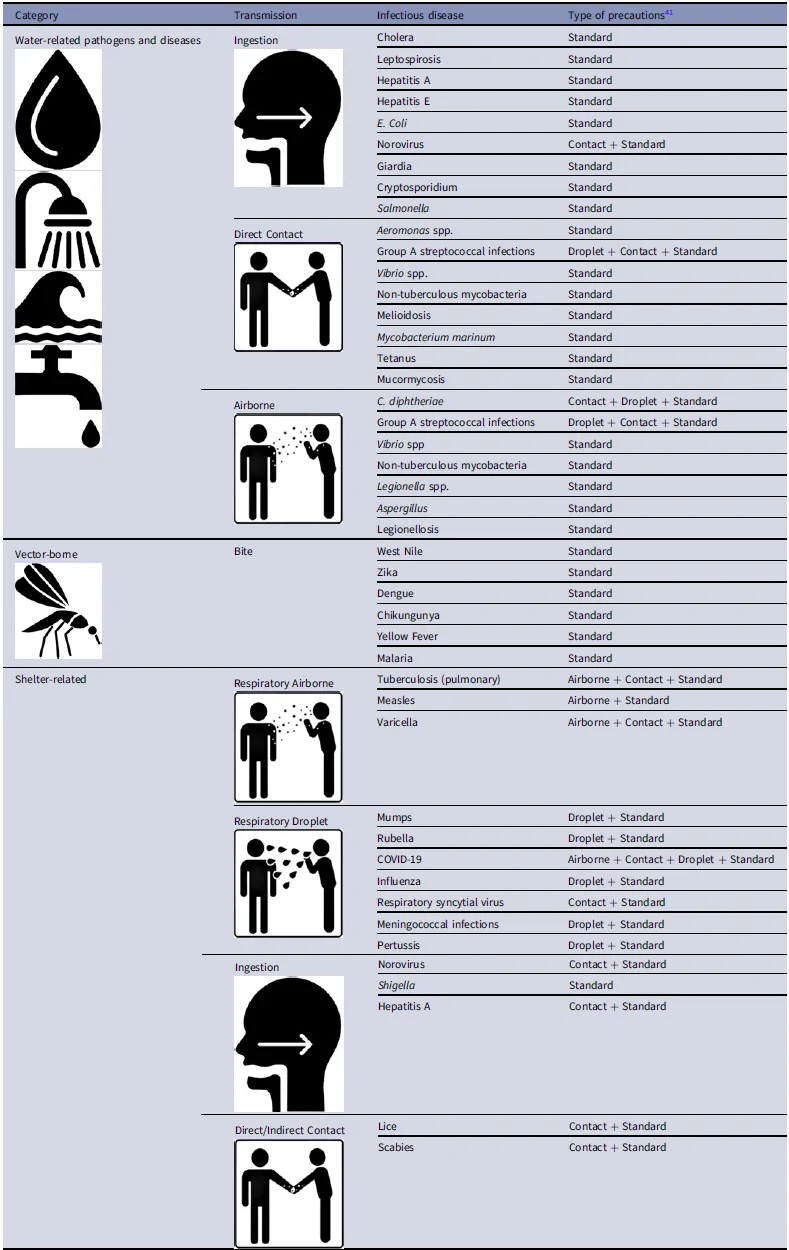
List of infectious diseases and corresponding transmission-based precautions Table 2 long description.

## Principles of infectious diseases in emergencies

Three fundamental principles should guide the response to and management of infectious diseases in emergencies: identify, isolate, and inform.^44^ The first step is to identify the presence of an infectious disease through clinical signs and symptoms as well as exposure history. Key epidemiological links and risk factors for transmission should be assessed, including crowding, inadequate sanitation, lack of potable water, and reduced access to health services. Once an infectious disease has been identified, the next step is to isolate the affected person(s) appropriately based on route of transmission to prevent further spread of disease. Use of PPE is based on the type of isolation precaution recommended for the disease of concern: standard, contact, droplet, and/or airborne.^43^ The final step is to inform the appropriate healthcare and infection prevention personnel immediately. In the US, preparedness and response includes mandatory reporting of certain infectious diseases to the local and/or state public health department. Effective bidirectional communication between healthcare facilities and health departments caring for survivors at local, state, and regional levels is also critical in managing communicable infectious diseases. Communication should also involve patients and their families, especially during the acute and recovery phases of the disaster.^45^

### Infectious disease surveillance

Early reporting and monitoring of disease trends is critical in disaster preparedness. It helps facilitate prompt detection and response to infectious disease outbreaks.^46^ This should be achieved in collaboration with local and/or state public health through existing systems in place to conduct epidemiologic surveillance. Daily review of hospital and clinic patient disease trends can also aid in recognizing signs of an impending outbreak.^46^

### Infectious diseases preparedness

Preparedness is essential for an effective outbreak response. Healthcare professionals and staff should be trained to recognize signs and symptoms of common infectious diseases. Readily accessible PPE, standardized infectious disease diagnostic and treatment protocols, stockpiling of essential medical supplies, and pre-established memorandums of understanding (MOU) to ensure rapid delivery of medications from suppliers during emergencies can further support an effective response. Prompt diagnosis and treatment are necessary for overall disease management. Pharmacies should maintain supplies of critical medications to treat or prevent infectious diseases including commonly used antimicrobials and vaccines. Medications to continue treatment of TB or HIV in patients with established infection should also be maintained. Medication use should be monitored daily and trended to alert when resupply is necessary. Regional contacts for medication distribution should be known by all and readily accessible to avoid critical shortages.^46^

Access to safe potable water is vital before and after a natural disaster. Facilities should arrange for a MOU to supply potable water for the immediate needs of the people and communities they serve. Sterile water for processing surgical instruments is crucial to ensuring that surgery can be performed safely. Likewise, environmental monitoring and control of humidity and temperature within healthcare facilities are necessary to maintain sterile surgical instruments and prevent healthcare-associated infections. Furthermore, facilities need to ensure they have plans and protocols to maintain operations during power outages.^47,48^

## Strategies for outbreak prevention

Strategies outlined below require health education and respect for health autonomy through improved disease recognition, self-promotion of hygiene and sanitation, and effective communication amongst all involved parties.^49^

### Access to food, clean water, and shelter

Several key factors must be considered to mitigate the risk of an infectious disease outbreak during and after a disaster, including shelter, water, sanitation, vector control, food and nutrition, immunizations, and health education.^49^ Temporary shelters, settlements, and camps for displaced survivors should have adequate living space to allow for distancing, functional waste disposal, adequate runoff drainage, proper ventilation, and physical security. There must be a safe and sufficient water supply, water system (ie, water tanks), efficient distribution of water, and proper waste control measures. Important questions to consider include water accessibility for drinking, cooking, showering, and flushing toilets. Sanitation is imperative through proper hand hygiene (including waterless alcohol products), waste control measures, shower head and faucet disinfectants, and decedent management. Vector control within shelters should entail use of personal protection (eg, clothing, insect repellant), residual spraying, environmental control, shelter design, and overall community awareness. Addressing the nutritional needs of affected communities is critical during emergencies. Malnutrition may lead to subsequent infections such as TB and compound diarrheal diseases.^49^

### Management of deceased individuals

There is no evidence that human remains cause infectious disease epidemics following disasters.^50^ However, except for known or suspected outbreaks of Ebola viral diseases during natural disasters, standard precautions should be taken when handling human remains or coming into contact with blood or other body fluids, including use of PPE, adherence to hand hygiene, and equipment disinfection. Guidance on PPE for handling the deceased individual due to viral hemorrhagic fever and Mpox is provided by the CDC.^51,52^ Individual and identifiable burials are preferred to mass cremation and mass graves; however, cultural and religious preferences need to be considered. Burial sites should be at least 200 meters away from water sources to prevent contamination.^53^

### Vaccination

Immunizations can be effective in preventing or mitigating infectious disease outbreaks. Disaster relief personnel must first define needs, identify target populations, plan vaccination strategies, implement campaigns, and evaluate at risk patients.^49^

## Impact of disasters on healthcare facilities

Disasters often result in widespread disruption and damage within healthcare facilities. Concurrent natural disasters may occur leading to further disruption of healthcare systems. The response by a US healthcare system during hurricanes Helene and Milton in 2024 provides details on the immense resources required for responding in real time.^54^

### Compromised power and water infrastructure

Loss of power can cause temperature extremes within healthcare facilities, leading to hyper- or hypothermia. Power outages also impact patient transport via elevators, patient safety via impaired connection to life-saving devices, and medication regulation via loss of pharmacy dispensaries as well as temperature-controlled medications, to name a few.^55^ These hospitals can also have water shortages and lose access to safe drinking water. Water shortages can lead to multiple challenges in healthcare facilities during natural disasters. Firstly, lack of clean water can limit hand hygiene leading to potential person-to-person transmission of disease. Water shortages can lead to inability to flush toilets creating unsanitary environmental conditions in the hospital. Furthermore, loss of access to safe drinking water increases risk for diarrheal diseases for patients. Finally, water shortage can lead to challenges with high-level disinfection of semi-critical items (eg, endoscopes) as well as sterilization of surgical instruments, limiting care and delivery of surgical services during natural disasters.^56^

### Staffing shortages and patient surges

Disasters also lead to staff shortages, due to staff not being able to travel to facilities or being directly impacted by a disaster themselves. Lack of transportation in general may lead to supply shortages, such as medications, food, and other necessary equipment and resources. These resource and healthcare personnel shortages are further exacerbated by increased patient volume and hospitalizations due to disaster-related injuries or other health events. Careful attention must be spent on evaluating patients for disaster-related infectious diseases, such as viral respiratory diseases or gastrointestinal diseases, that may be easily transmitted (eg, norovirus) and require appropriate and timely isolation.^57^

### Impacts of water intrusion on healthcare delivery

In addition to the concerns above, water intrusion due to flood water may lead to contamination of the environment with pathogens, human/animal waste, and hazardous chemicals. Wet areas, unless properly remediated, may lead to fungal growth with risks for infection (especially among immunocompromised patients) and/or respiratory ailments (eg, asthma). Water intrusion may also lead to electrical hazards, damage key equipment, and impair all healthcare services.^25^

## Management of prevalent and chronic infectious diseases

Management of certain prevalent diseases including TB, HIV, HBV, and HCV require urgent management and response during and postdisasters. TB is one of the leading infectious causes of death globally, with an estimated incidence of 10 million people globally in 2020.^58^ During acute and postacute recovery, patients with TB will require continued access to anti-TB medications per established national and international World Health Organization (WHO) guidelines to limit transmission and reduce risk of developing resistant disease.^59,60^ HIV, with an estimated 41 million people affected worldwide, requires similar coordination and response.^61^ Standard treatment regimens should follow WHO, US Department of Health and Human Services, National Institute of Health, and International Antiviral Society—United States of America.^62–65^ In disaster situations where medications are scarce, antiviral medications can be switched temporarily to minimize interruptions in treatment.^66^ In 2022, it was estimated that 304 million people were living with chronic HBV or HCV globally, and roughly 1.3 million people die annually from these viruses.^67^ Standard treatment guidelines for HBV and HCV are provided by the WHO.^67,68^

## Additional considerations for planning and preparedness

Disaster impacts are diverse, including healthcare effects such as product shortages. Below are some additional disaster outbreaks, shortages, guidelines, and resources to consider. Hurricane Helene caused widespread intravenous fluid supply and peritoneal dialysis solution shortages after major flooding shut down local factory production and distribution.^69,70^ During COVID-19, the demand for oxygen left many low- and middle-income countries vulnerable to oxygen tank shortages, due to cost, infrastructure, and accessibility.^71^ Blood culture bottles remain in low supply, leading to potential delays in diagnosis, misdiagnosis, and other healthcare challenges.^72^

The US Food and Drug Administration provide real time information on existing drug shortages and medical device shortages to better plan, prepare, and respond in the face of disasters.^73,74^ The Society for Healthcare Epidemiology for America (SHEA) provides education, guidance, and resources for emerging infectious threats.^75,76^ Preparedness also includes monitoring international settings of outbreaks as provided by the WHO.^77^

## Conclusion

*By failing to plan, you are planning to fail.* (Benjamin Franklin)^78^

Disasters are widespread, impacting communities in many ways. Accordingly, healthcare systems must stay alert to both existing and emerging threats, with a strong focus on emergency preparedness across critical domains, including supply chains, access to care, and workforce capacity. Ultimately, strengthening resilience at the individual, environmental, and community levels is essential to support effective preparedness and improve recovery across the disaster continuum.

## Research transparency and reproducibility

The authors did not use any data sets, codes, materials, or processes necessary for others to fully evaluate the basis for the findings in this manuscript. They did use information from several public resources including ACEP, CDC, FEMA, WHO, and NETEC.

## Table 1. Long description

A table with five columns: Category, Definition, Information, and Disaster-related examples. The table has 8 rows, each detailing different types of injuries and diseases. Row 1: Drowning, defined as respiratory impairment from liquid submersion, with 300,000 annual deaths, highest in children under 5, and common in flood disasters. Row 2: Burn injuries, caused by heat or chemicals, with 180,000 annual deaths, high morbidity, and more child deaths in low- and middle-income countries. Row 3: Blunt force trauma, leading cause of death worldwide, includes crush injuries. Row 4: Bites, 5 million snake bites annually with 50 percent envenomation, causing paralysis and other severe complications. Row 5: Carbon monoxide poisoning, symptoms include headache and dizziness, can lead to coma and death. Row 6: Electrocution, causes death by electrical injury, with higher voltage causing severe burns and death. Row 7: Medical, chronic conditions requiring ongoing care, disrupted by disasters. Row 8: Psychiatric, significant disturbance in cognition or behavior, 70 percent of people experience traumatic events, 4 percent have PTSD

Navigate back to Table 1..

## Table 2. Long description

The table lists various infectious diseases and the types of precautions required for each, categorized by pathogens and diseases, bite, and shelter-related infections. Each disease is associated with a type of precaution such as Standard, Contact, Droplet, Airborne, etc.

Navigate back to Table 2..
